# Targeting the “PVR–TIGIT axis” with immune checkpoint therapies

**DOI:** 10.12688/f1000research.22877.1

**Published:** 2020-05-13

**Authors:** Laurent Gorvel, Daniel Olive

**Affiliations:** 1 Cancer Research Center of Marseille, INSERM U1068, CNRS U7258, Aix Marseille Université, Institut Paoli – Calmettes, Marseille, France

**Keywords:** PVR, Nectin2, DNAM-1, TIGIT, CD96, Immune checkpoint therapy

## Abstract

Checkpoint inhibitors have become an efficient way to treat cancers. Indeed, anti-CTLA-4, anti-PD1, and anti-PDL-1 antibodies are now used as therapies for cancers. However, while these therapies are very efficient in certain tumors, they remain poorly efficient in others. This might be explained by the immune infiltrate, the expression of target molecules, and the influence of the tumor microenvironment. It is therefore critical to identify checkpoint antigens that represent alternative targets for immunotherapies. PVR-like molecules play regulatory roles in immune cell functions. These proteins are expressed by different cell types and have been shown to be upregulated in various malignancies. PVR and Nectin-2 are expressed by tumor cells as well as myeloid cells, while TIGIT, CD96, and DNAM-1 are expressed on effector lymphoid cells. PVR is able to bind DNAM-1, CD96, and TIGIT, which results in two distinct profiles of effector cell activation. Indeed, while binding to DNAM-1 induces the release of cytokines and cytotoxicity of cytotoxic effector cells, binding TIGIT induces an immunosuppressive and non-cytotoxic profile. PVR is also able to bind CD96, which induces an immunosuppressive response in murine models. Unfortunately, in humans, results remain contradictory, and this interaction might induce the activation or the suppression of the immune response. Similarly, Nectin-2 was shown to bind TIGIT and to induce regulatory profiles in effectors cells such as NK and T cells. Therefore, these data highlight the potential of each of the molecules of the “PVR–TIGIT axis” as a potential target for immune checkpoint therapy. However, many questions remain to be answered to fully understand the mechanisms of this synapse, in particular for human CD96 and Nectin-2, which are still understudied. Here, we review the recent advances in “PVR–TIGIT axis” research and discuss the potential of targeting this axis by checkpoint immunotherapies.

## Introduction

Methods to stimulate the immune system and target malignant cells have evolved over several decades. Indeed, numerous studies showed that patients can develop immune responses against tumor antigens, although clinical benefits remained weak. The reasons for this rely on factors such as the immunogenicity of the target antigen and the tumor microenvironment. On the one hand, target antigens play a critical part in the immunogenicity of a vaccine. On the other hand, the tumor microenvironment has repeatedly been shown to interfere with immune cell function. T cell checkpoint inhibitors have become an effective way to treat cancers. Indeed, antagonistic antibodies, like anti-CTLA-4, are now used as therapies in several cancers and led the way for the targeting of similar immunoregulatory molecules such as programmed cell death (PD-)1 and PD ligand-1 (PDL-1). Anti-PD1 and PDL-1 antibodies are now approved by the US Food and Drug Administration to be used as therapeutic agents ^1^. However, they are efficient in some cancer types and remain inefficient in others. It is therefore critical to identify checkpoint antigens that represent alternative and/or better targets for immunotherapies.

**Figure 1.**
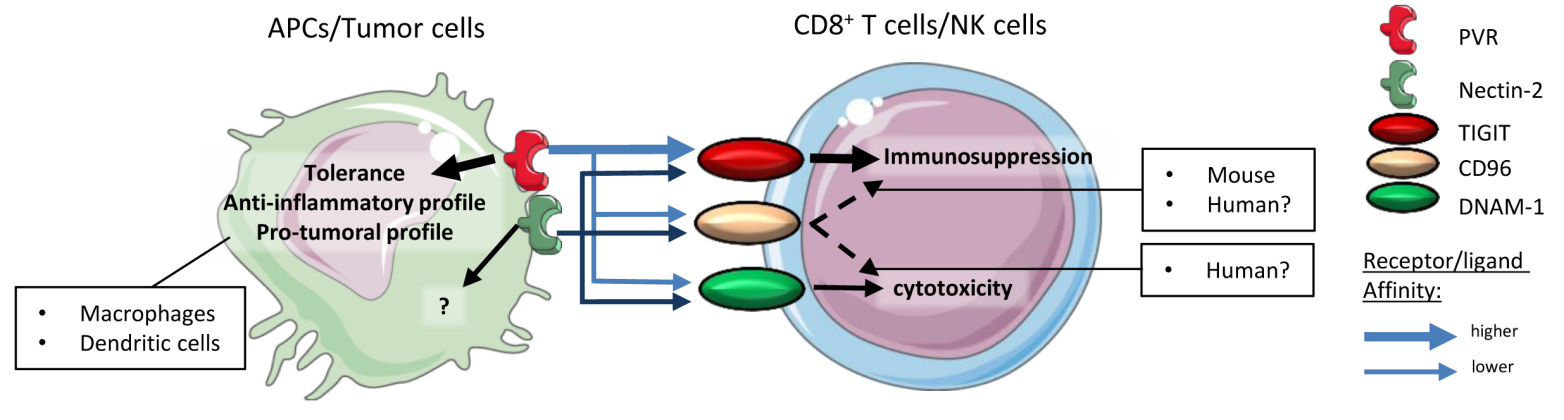
The PVR–TIGIT axis. PVR and Nectin-2 are expressed on APCs or tumor cells. TIGIT, CD96, and DNAM-1 are expressed on cytotoxic effector cells (CD8 ^+^ T cells and NK cells). PVR affinity for TIGIT is higher than its affinity for CD96 or DNAM-1. Thus, the signaling of the PVR–TIGIT synapse induces immunosuppression rather than effector cell activation and/or cytotoxicity. Signaling through PVR induces anti-inflammatory profiles in dendritic cells and macrophages. CD96 signaling induces immunosuppression in murine models, which was not demonstrated in human models. Similar to PVR, Nectin-2 binds PVR, CD96, or DNAM-1 but with a lower affinity than PVR. APC, antigen-presenting cell; DNAM-1, DNAX accessory molecule-1; NK, natural killer; PVR, poliovirus receptor; TIGIT, T Cell Immunoreceptor with Ig and ITIM domains.

The poliovirus receptor (PVR)-like molecules are a group of the immunoglobulin superfamily that play regulatory roles in T cell and NK cell functions. PVR-like molecules share PVR-signature motifs in the first immunoglobulin variable-like domain and are originally known to mediate epithelial cell–cell contact. PVR (also known as CD155 or Necl-5) and Nectin-2 (also known as Pvrl2 or CD112) are two major ligands that are expressed on epithelial and myeloid cells of the tumor (Human Cell Atlas, ^2, 3^). PVR is an integral membrane protein that binds poliovirus and is of interest because it is upregulated during the response to DNA damage, a process that occurs in viral infections and cancers. PVR is able to bind CD226, DNAX accessory molecule-1 (DNAM-1), T Cell-Activated Increased Late Expression Protein, TACTILE (CD96), and T Cell Immunoreceptor with Ig and ITIM domains (TIGIT), which results in a very distinct profile of effector lymphoid cell activation. While binding to DNAM-1 induces the release of pro-inflammatory cytokines and cytotoxicity of T cells and NK cells, binding TIGIT induces a rather anti-inflammatory, non-proliferative, and non-cytotoxic profile ^4– 6^. Similarly, Nectin-2, an adhesion protein, was shown to bind TIGIT and to induce regulatory profiles in effector cells such as NK and T cells ( Figure 1). A recent study by Stamm *et al.* showed that the use of an anti-PVR or anti-TIGIT monoclonal antibody (mAb) resulted in increased lysis of breast cancer cell lines by cytokine-induced killer cells ^7^. Therefore, PVR, TIGIT, and CD96 also represent interesting targets for immunotherapies because of their expression on the lymphoid effector cells and their immunoregulatory function and involvement in various cancers ^3, 8^.

Here we will review the recent advances in PVR–TIGIT axis research and discuss the potential of targeting this axis with immunotherapies. First, we will discuss the expression and function of PVR and Nectin-2 in the modulation of the immune system. Second, we will discuss the expression and function of TIGIT, DNAM-1, and CD96 on lymphoid effector cells as well as tumor cells. Altogether, the aim of this review is to give a comprehensive overview of the interactions between the players of the “PVR–TIGIT synapse” and assess their potential as immunotherapy targets.

## Function of PVR and Nectin-2 in the regulation of the immune response

### PVR as a relevant new target for immunotherapy

**Figure 2.**
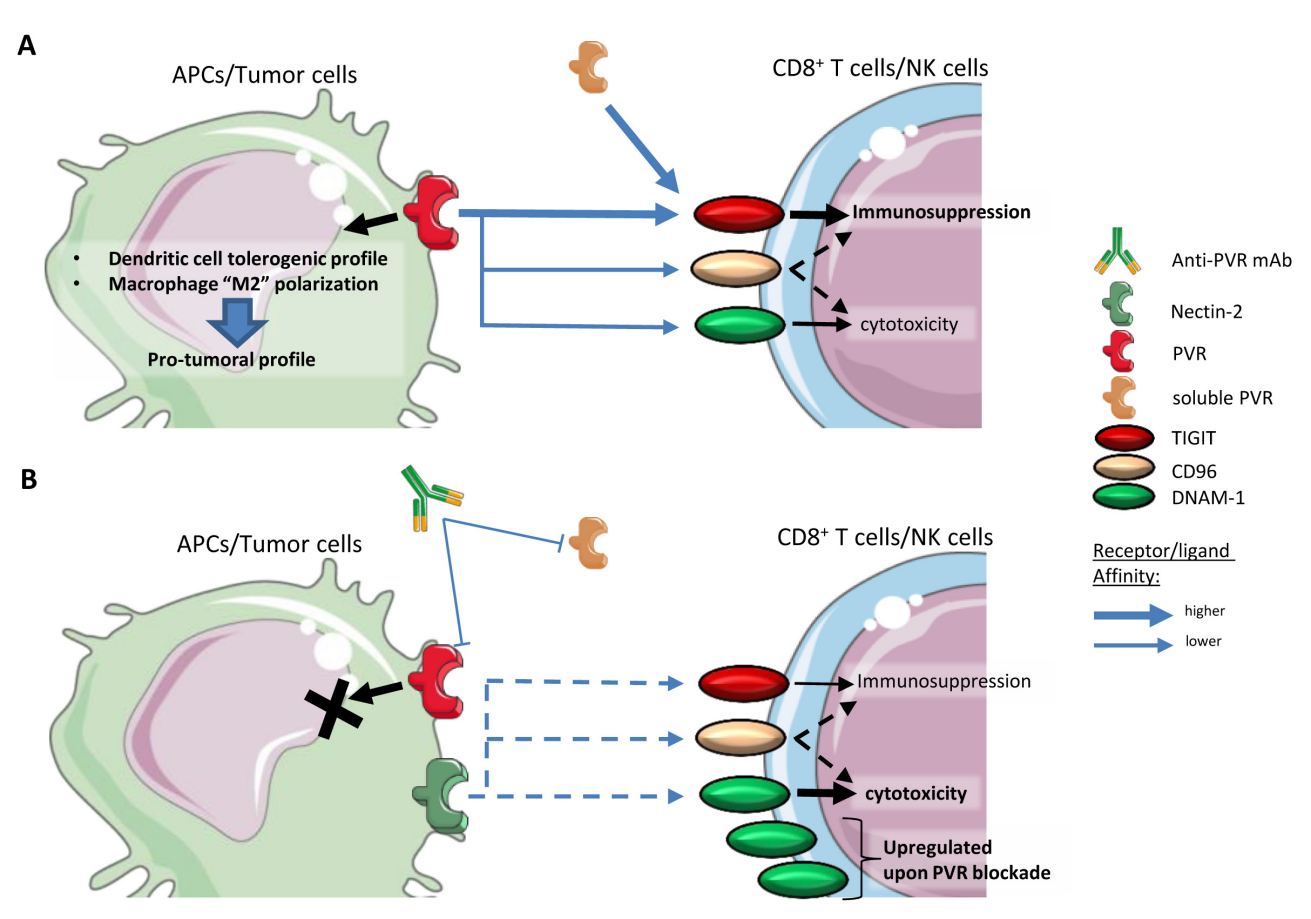
PVR’s role in immunomodulation. **A**. Classical role of PVR in synapse signaling, tweaking the immune response toward a pro-tumoral profile. **B**. Using an antagonistic mAb directed against PVR would block its interaction with TIGIT, CD96, and DNAM-1. This would partially inhibit the signaling pathway, as the synapse would be maintained by Nectin-2 but with a much weaker affinity. Upon blockade, DNAM-1 expression would be upregulated in effector cells. APC, antigen-presenting cell; DNAM-1, DNAX accessory molecule-1; mAb, monoclonal antibody; NK, natural killer; PVR, poliovirus receptor; TIGIT, T Cell Immunoreceptor with Ig and ITIM domains.

PVR (CD155) was shown to be the poliovirus’s point of entry into cells, hence its name. It is a cell adhesion molecule that allows adhesion and/or migration following a gradient of chemoattractant ^9^. Indeed, staining demonstrated that PVR accumulates at the edges of lamellipods, pseudopods, or dendrites ^9^. PVR expression was associated with an unfavorable prognosis in solid tumors such as colon cancer, breast cancer, lung adenocarcinoma, pancreatic cancer, melanoma, and glioblastoma, as it correlated with tumor migration, development of metastases, tissue and lymph node invasion, relapse, and poorer survival ^10– 15^. PVR was demonstrated to be upregulated upon DNA damage after signaling through the Sonic hedgehog pathway or after stimulation of the RAS and TLR4 pathways. This is relevant for cancer therapy, as chemotherapy might induce the expression of PVR and therefore either improve immune response or increase immunosuppression ^9, 16^. This comes from the fact that PVR binds to three different molecules, which leads to very different outcomes. Indeed, PVR might bind to DNAM-1 (which is expressed on NK cells and cytotoxic CD8 ^+^ T cells) and deliver a positive signal, leading to an anti-tumoral response (see DNAM-1 section). However, PVR preferentially binds TIGIT, for which it has more affinity, and therefore tends to induce an immunosuppressive profile of TIGIT-expressing cells. PVR was also described to bind CD96, whose function remains elusive in humans (see CD96 section). Interestingly, the reverse signal following PVR binding to DNAM-1 or TIGIT was not extensively studied, and only a few studies demonstrated that this signal through PVR influences the polarization of macrophages toward an anti-inflammatory M2-like profile ^17^. Similarly, the signaling through PVR in dendritic cells induced a rather tolerogenic profile. Indeed, the use of recombinant human TIGIT-fc fragments allowed mature dendritic cells to produce IL-10 and regulated the expression of activation markers such as CD80 and CD86 ^18^ ( Figure 2, A). This immunosuppressive effect might be due to the presence of an ITIM motif in the isoform of PVR. Noteworthily, PVR exists in four different isoforms: α and δ are transmembrane, and δ lacks the ITIM motif, and β and γ are soluble isoforms that lack the transmembrane domains ^9^. We can hypothesize that this effect is a negative feedback signal on antigen-presenting cells to avoid unnecessary inflammation.

However, in the case of cancer, such negative signaling is of interest, and the idea of blocking PVR becomes attractive. Indeed, blocking PVR would result in blocking three ligands (TIGIT, DNAM-1, and CD96). Considering that PVR has a better affinity for the immunosuppressive TIGIT, this could be enough to reverse immunosuppression. This would also allow the interaction of DNAM-1 with its other ligand Nectin-2 and promote an anti-tumoral response. However, TIGIT is also able to bind Nectin-2 and could still maintain immunosuppression (see Nectin-2 section). Interestingly, PVR blockade might be beneficial for the expression of DNAM-1, as studies demonstrated that the expression of PVR downregulates that of DNAM-1 and that blocking PVR would result in increased levels of DNAM-1 ^9, 19^. In support of a PVR blockade strategy, studies in acute myeloid leukemia (AML) and melanoma cell lines as well as *in vivo* murine tumor models resulted, respectively, in the activation of anti-tumoral cell types and avoidance of metastases ( Figure 2, B). Noteworthily, clinical trials targeting PVR and its interactions are either antibodies directed against TIGIT in combination with other mAbs or chemotherapy or radiation (refer to TIGIT section) or use PVR as a point of entry for recombinant oncolytic polioviruses in the case of advanced gliomas (NCT01491893, NCT03043391, and NCT02986178) or triple negative breast cancer (NCT03564782) ^9^.

Altogether, PVR represents a potential immunotherapy target thanks to its role as a keystone of the PVR–TIGIT immunosuppressive synapse.

### Is Nectin-2 a good target for immunotherapies?

Nectins are cell adhesion molecules which are involved in various developmental processes including but not limited to ear development, spermatogenesis, myelination, and axon guidance ^20^. They also were described to be involved in neurodegenerative diseases such as Alzheimer’s disease, viral infections, and cancers. Nectin-2 was shown to be expressed in breast and ovarian tumors ^20, 21^. Recently, their involvement in immune response modulation was proved, as they were part of the PVR-like signaling pathway. More specifically, Nectin-2 (PRR2, PVRL2, and CD112), a protein expressed by primary tumor cells, cell lines, and to a lesser extent on immune cells such as monocytes, macrophages, B cells, and dendritic cells, was shown to interact with DNAM-1 and/or TIGIT to alter the immune response (Human Cell Atlas, ^9, 13^). Indeed, similar to PVR, Nectin-2 is able to bind DNAM-1 on CD8 ^+^ T cells and NK cells, leading to an anti-tumoral response, or TIGIT, leading to a pro-tumoral response via the inhibition of cytotoxicity. However, while PVR binds with a strong affinity to TIGIT, Nectin-2 affinity to TIGIT is much weaker. Considering this redundancy with PVR function in the modulation of the immune response, Nectin-2 could be an interesting target for immunotherapy as well. Indeed, using antagonistic mAbs to block Nectin-2 interaction with TIGIT could represent an alternative in the cases where PVR is weakly expressed. Furthermore, no reverse signaling through Nectin-2 has been demonstrated to date and therefore would not affect a potential beneficial response induced by Nectin-2 binding to its ligands. Interestingly, Nectin-2 was shown to bind PVR Related Immunoglobulin Domain Containing (PVRIG), another inhibitory receptor of the nectin family. The blockade of PVRIG using a mAb resulted in increased production of cytokines and cytotoxicity of CD8 ^+^ T cells, which was not the case after TIGIT blockade ^22^. This highlighted that Nectin-2 inhibition was mainly dependent on its binding to PVRIG. To our knowledge, no clinical trials involving the targeting of Nectin-2 are currently ongoing.

## TIGIT, DNAM-1, and CD96 function in effector cells

### The good: DNAM-1

DNAM-1 is expressed by a highly diverse set of immune cells, which includes T cells (CD8 ^+^ and CD4 ^+^), NK cells, B cells, and monocytes. Its function has been studied intensively in NK cells and CD8 ^+^ T cells, where its activation leads to the activation of these cytotoxic cells as well as their degranulation and therefore stimulates their anti-tumoral response ^23, 24^. Therefore, it is not surprising to find that DNAM-1 expression is downregulated in various types of tumors including ovarian and breast cancer ^14, 25, 26^. DNAM-1’s strong activation functions allowed the development of a rationale to target or use this molecule in the treatment of malignancies. Indeed, ideas of using DNAM-1 ^+^ cytokine-induced killer cells or developing DNAM-1 chimeric antigen receptor (CAR) T cells have emerged ^27, 28^. Creating autologous T cells which are positive for DNAM-1 would increase their efficiency and specificity in the case of PVR ^+^ and Nectin-2 ^+^ tumors. Interestingly, no clinical trials are currently ongoing with agonistic mAbs directed against DNAM-1 to stimulate CD8 ^+^ T cells and NK cells, which is probably because of the broad expression of DNAM-1 among immune cells and therefore the increasing chance of observing adverse effects.

### The bad: TIGIT, a rising star in immune checkpoint targeting

**Figure 3.**
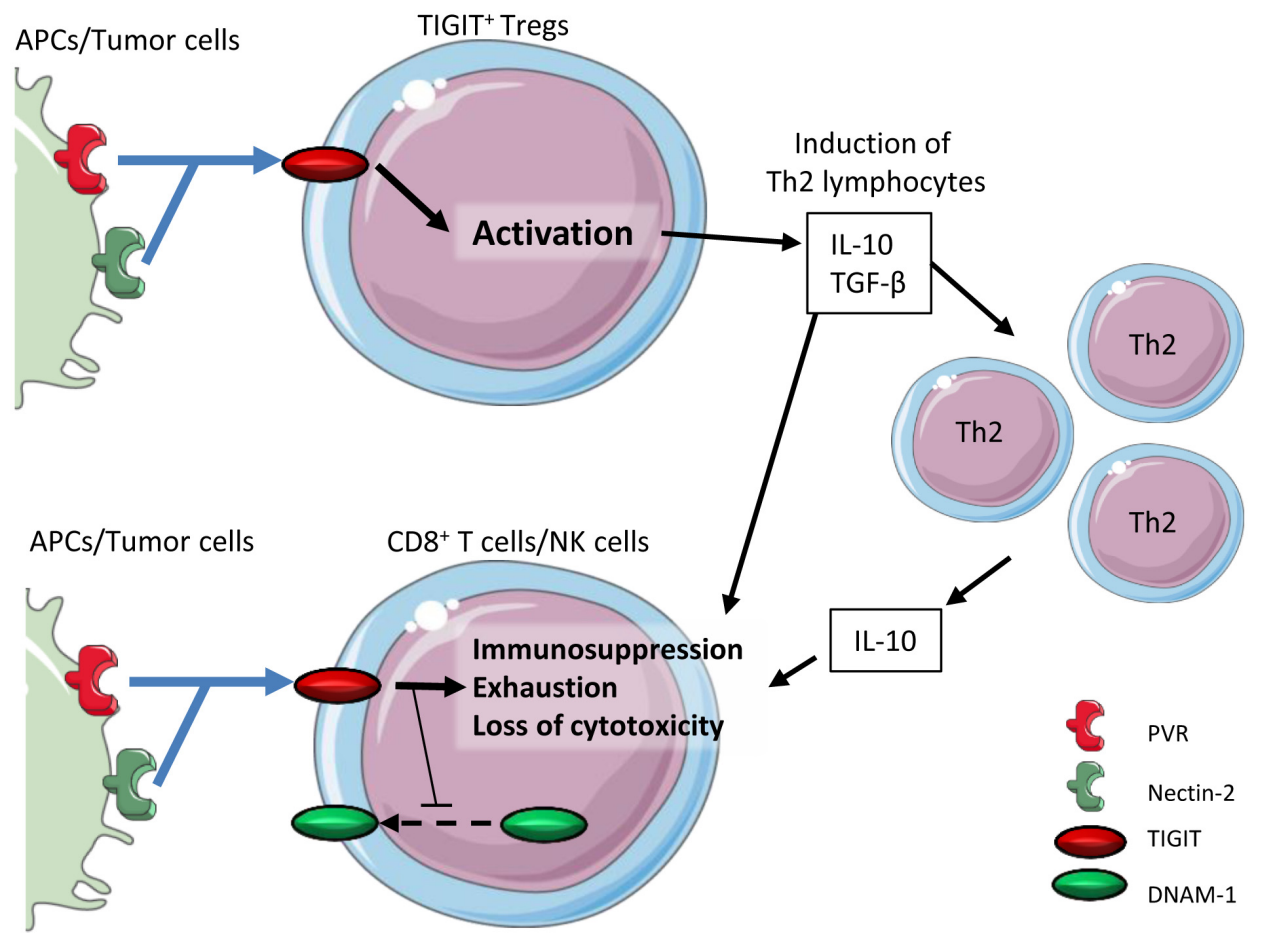
TIGIT immunosuppressive function. TIGIT binds to its ligand, which induces the production of IL-10 and TGF-β by Tregs. This induces the polarization of T cells toward a Th2 profile, thus avoiding inflammation. IL-10 will create an anti-inflammatory microenvironment, which participates in the inhibition of CD8 ^+^ T cell cytotoxicity. The binding of TIGIT by its ligands induces a loss of cytotoxicity, immunosuppression, and exhaustion on effector cytotoxic cells. Signaling through TIGIT also blocks DNAM-1 dimerization, which leads to decreased DNAM-1 expression on effector cells. APC, antigen-presenting cell; DNAM-1, DNAX accessory molecule-1; IL, interleukin; PVR, poliovirus receptor; TGF, transforming growth factor; TIGIT, T Cell Immunoreceptor with Ig and ITIM domains; Th2, T helper type 2; Treg, regulatory T cell.

The number of studies focusing on TIGIT has increased over the past few years and allowed researchers to form a catalog of TIGIT expression in tumors. Indeed, TIGIT was found to be expressed on the lymphoid cells in non-small cell lung carcinoma (NSCLC), melanoma, breast cancer, colon adenocarcinoma (COAD), AML, and multiple myeloma (MM) ^4, 29– 32^. TIGIT is an inhibitory immunoglobulin receptor which possesses a tyrosine-based inhibitory motif domain (ITIM). It is expressed by CD8 ^+^, CD4 ^+^ T cells (mainly regulatory T cells [Tregs]), and NK cells ^18, 33^. It represents the immunosuppressive signaling counterpart of DNAM-1, with which it competes for the ligation of Nectin-2 and PVR. However, TIGIT was described to present more binding affinity for PVR than DNAM-1, which means that the expression of TIGIT on tumor-infiltrating lymphocytes is powerful enough to skew the immune response toward an immunosuppressed phenotype and therefore abrogates NK cell and CD8 ^+^ T cell cytotoxicity ^33^. Furthermore, TIGIT signaling was also described to prevent the homodimerization of DNAM-1, therefore avoiding its signaling and the subsequent antitumoral response. TIGIT was also associated with PD-1 expression, among a 15-gene signature which was related to T cell exhaustion ^5, 34^. Interestingly, TIGIT is expressed on a Treg subset, which is surprising considering that signaling through TIGIT usually inhibits the function of the cell type it is expressed on. Therefore, Tregs which express TIGIT could be less immunosuppressive. A response was given by Bottino *et al.* that described the functional role of TIGIT ^+^ Tregs compared to their negative counterpart. They demonstrated that TIGIT ^+^ Tregs skewed the CD4 ^+^ T cell response toward a Th2 profile, therefore suppressing the function of Th1 inflammatory cell types ^35^. In addition, *in vivo*, TIGIT activation led to the suppression of CD8 ^+^ T cell cytotoxicity via the production of IL-10 ( Figure 3).

Taken together, these findings highlight the powerful immunosuppressive functions of TIGIT. Indeed, the expression of TIGIT on cytotoxic cells prevents their activation and degranulation by competition with DNAM-1, while its expression on Tregs allows the suppression of other inflammatory cell types via the regulation of anti-inflammatory cytokines. This is why targeting TIGIT with antagonistic mAbs appears to be a logical strategy. So far, six clinical trials using anti-TIGIT antibodies are ongoing. Oncomed Pharmaceutical’s anti-TIGIT mAb etigilimab (OMP-313M32) underwent safety and pharmacodynamics testing in a phase I dose escalation study (NCT031119428) alone or in combination with anti-PD1 (nivolumab) in an attempt to treat advanced solid tumors. Etigilimab seemed to be well tolerated by patients after phase Ia; however, this trial was stopped in phase Ib for sponsor reasons. Other phase I and II clinical trials are still ongoing: tiragolumab (Genentech Roche) for the treatment of NSCLC, AB154 (Arcus Biosciences) for the treatment of advanced solid malignancies, MK-7684 (Merck) for the treatment of solid tumors, BMS986207 (Bristol Myers Squibb) for the treatment of advanced and metastatic solid tumors, and ASP8374 (Astellas Pharma, Potenza Therapeutics) for the treatment of advanced or metastatic solid malignancies (reviewed in 29).

### The ugly: the role for human CD96 in immune response modulation is poorly understood

**Figure 4.**
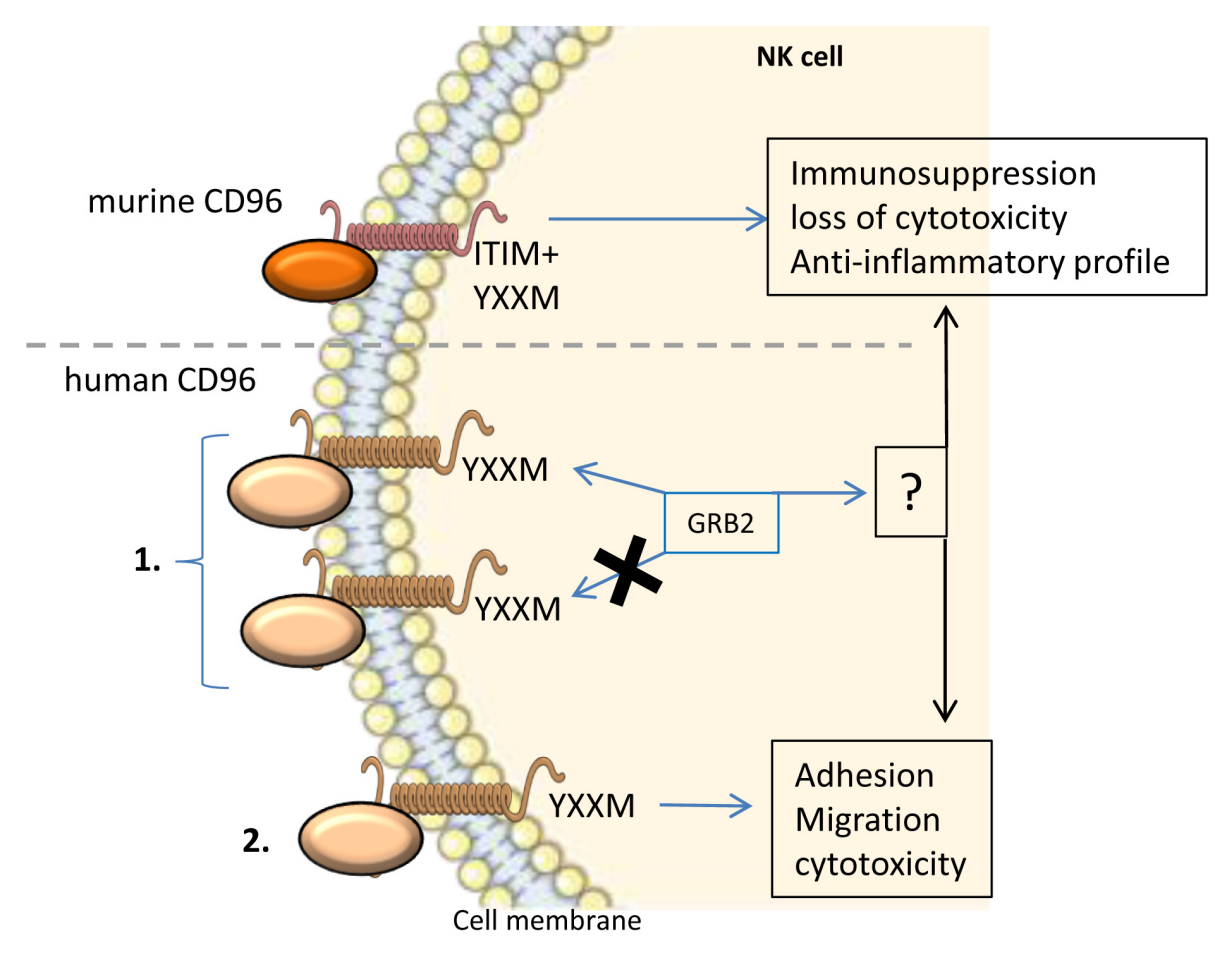
Murine and human CD96 signaling and function in NK cells. Murine CD96 possesses an intracellular ITIM motif, which leads to an inhibitory signal and the abrogation of the inflammatory immune response and cytotoxicity of NK cells. Human CD96 signaling is more complex and poorly studied. 1. The intracellular domain of human CD96 harbors a YXXM motif, which may or may not bind GRB2. GRB2 might lead to the activation or inhibition of NK cells. 2. Human CD96 signaling was shown to induce adhesion, migration, and cytotoxicity in NK cells. GRB2, growth factor receptor-bound protein 2; ITIM, immunoreceptor tyrosine-based inhibition motif; NK, natural killer.

CD96 was first described as a marker of T cells (TACTILE); however, besides its expression, there was no supporting evidence of a strong functional aspect of CD96 in these cells. CD96 is also expressed on NK cells, in which, in murine models, signaling through CD96 would inhibit inflammatory cytokine production as well as cytotoxicity ^36^. Still, in murine models, CD96 was proven to possess a direct inhibitory role of anti-tumor function notably in NK cells. Indeed, CD96 inhibits IFN-γ production by NK cells, which would have interacted with mature/activated dendritic cells. In support of this information, anti-mCD96 mAb increased metastasis reduction after tumor resection. However, CD96 in humans exhibits a different profile, and a direct inhibition mechanism through CD96 could not be demonstrated ^14, 37^. Therefore, multiple hypotheses are being investigated. Among them, one proposition is that the inhibition of an anti-tumoral response is compartmentalized between TIGIT and CD96. In this hypothesis, TIGIT would mainly inhibit CD8 ^+^ T cells while CD96 would inhibit NK cells. However, it was described that the engagement of human CD96 could activate NK cells, notably by inducing adhesion, promoting cytotoxicity and PVR acquisition of target cells ^38^. This highlights the fact that CD96 signaling might be more complex than just an inhibitory pathway. Following this idea, an investigation of the intracytoplasmic domain of CD96 is necessary to understand its signaling and function. CD96 exhibits a short intra-cytoplasmic tail that may bind to GRB2 depending on the presence or the absence of a YXXM motif ^39, 40^. YXXM motifs were shown to bind and activate phosphatidylinositol 3-kinases (PI3Ks) and subsequently the AKT kinase ^41– 43^. A YXXM motif and downstream signal were shown to regulate growth, proliferation, survival, and immune response regulation. Also, the PI3K pathway was upregulated in most human tumors and found to be involved in cancer cell resistance to anti-tumor therapies ^43– 45^. Interestingly, a YXXM motif-bearing short cytoplasmic tail was also found in the cases of CD28 as well as ICOS signaling and revealed two outcomes. Indeed, in the case of CD28 signaling, the short cytoplasmic tail binds to GRB2, therefore inducing the production of IL-2. On the contrary, the ICOS short cytoplasmic tail cannot bind GRB2, which stops the signaling and therefore did not lead to IL-2 production. However, the importance of the YXXM motif of human CD96 for signaling and response cannot be assessed owing to its very low specificity ^6, 39^ ( Figure 4). Finally, it remains possible that the response to stimulation of human CD96 might depend on the cell type that it is expressed on. This means that the use of anti-human CD96 mAbs should be carefully considered and that immunophenotyping should be performed for each patient to determine the expression pattern of CD96.

## Concluding remarks

The immunological synapse formed by the interaction of PVR and Nectin-2 with their receptors DNAM-1, TIGIT, and CD96 represents a complex interaction system. These interactions are being increasingly studied by the scientific community, as they are being included in a global effort to understand the dynamics and the function of putative immunotherapy targets. The PVR–TIGIT axis is composed of two main ligands (PVR and Nectin-2), which interact with three receptors (DNAM-1, TIGIT, and CD96). Therefore, designing an approach for targeting any of these molecules with antagonistic mAbs should consider the multiple interactions and alternative binding that may occur and avoid an optimal response. Indeed, blocking TIGIT interactions with its ligands seems obvious considering the inhibitory nature of its signaling. However, one should consider that CD96, as ambiguous as its functions seem to be in humans, might participate in the inhibition of an anti-tumor response. Similarly, as mentioned in the section dedicated to PVR, blocking PVR might not be enough to rescue a fully active anti-tumoral response because Nectin-2 is able to bind TIGIT too and induce an inhibitory signal. PVR is receiving more and more attention as a checkpoint blockade therapy target, mainly for its keystone role in the PVR–TIGIT axis. Indeed, PVR is able to bind both inhibitory and activating receptors of the nectin-like protein family, and studies show that using anti-PVR mAbs to avoid its interactions with inhibitory receptors allows the recovery of the anti-tumoral response ^9, 19, 46^. This makes PVR an interesting checkpoint blockade target. In addition, PVR is being evaluated in clinical trials as a point of entry for oncolytic polioviruses (see PVR section). DNAM-1 is also being investigated as a target for immunotherapy considering its function in the activation of cytotoxic lymphocytes. Indeed, strategies aimed at stimulating the DNAM-1 pathway are being developed, including the use of agonistic mAbs or DNAM-1-based adoptive cell transfer. Nectin-2 remains poorly studied as a target for immunotherapy, mainly because of its weaker affinity for both DNAM-1 and TIGIT, as well as the absence of retro-signal. However, Nectin-2 still represents a potential target, especially in tumors where its expression is elevated. CD96 could also represent an obvious target for immune checkpoint blockade if its function was as clear in humans as it is in murine models. Indeed, while murine CD96 signaling induces an immunosuppressive signal, it is less clear in humans where CD96 might be an activator or an inhibitor, which might also depend on the cell type they are expressed on. Therefore, more thorough studies on the regulation, signaling, and function of CD96 might be necessary to evaluate the true potential of CD96 as an immune checkpoint therapy target. Nonetheless, the PVR–TIGIT synapse represents a keystone in the regulation of the immune response to the tumoral microenvironment and offers multiple angles of attack to rescue a proper anti-tumoral response.
